# A literature-based cost-effectiveness analysis of device-assisted suturing versus needle-driven suturing during laparotomy closure

**DOI:** 10.1007/s10029-025-03266-2

**Published:** 2025-01-23

**Authors:** Zin Min Thet Lwin, Gabriel Börner, Sophia Verheij-Engqvist, George Keel

**Affiliations:** 1 Department of Molecular Medicine and Surgery, Karolinska Institutet, Karolinska University Hospital, Solna (L1:00), Stockholm, 171 76 Sweden; 2 The Swedish Institute for Health Economics, Svartmangatan 18, Stockholm, 111 29 Sweden; 3 Department of Clinical Sciences, Faculty of Medicine, Lund University, Box 50332, Lund, Malmö, 202 13 Sweden; 4 Suturion AB, Scheeletorget 1, Lund, 223 63 Sweden; 5 Department of Learning, Informatics, Management, and Ethics, Karolinska Institutet, Tomtebodavägen 18A, Stockholm, 171 65 Sweden

**Keywords:** Laparotomy, Sutures, Cost-effectiveness analysis, Surgical equipment

## Abstract

**Purpose:**

Small-bites suturing technique for laparotomy closure is now recommended as the standard of care. However, uptake of the practice remains slow. A medical technology called the SutureTOOL has been developed which can facilitate implementation of small-bites. The aim of the study was to compare the economic and clinical outcomes of laparotomy closure for patients using manual needle-driver suturing versus device-assisted suturing (SutureTOOL) following open abdominal surgery.

**Methods:**

This cost-effectiveness analysis comparing device-assisted suturing to needle-driver suturing was performed from a healthcare perspective within Sweden, France, the UK, and the US. A decision tree model was developed to implement the analysis.

**Results:**

The SutureTOOL was found to be cost-effective, reducing costs between 22% and 40% across country contexts. Savings were associated with reduced post-operative complications and reductions in operating room time. Improvements in quality of life were minimal and not clinically significant, likely because of the short time horizon.

**Conclusion:**

Cost-effectiveness was largely due to cost savings. Prior to procurement, hospitals should test the device to ensure that small-bite rates and reductions in operation time are replicable within their clinical context. If so, the device will improve quality of care for laparotomy wound closure.

**Supplementary Information:**

The online version contains supplementary material available at 10.1007/s10029-025-03266-2.

## Introduction

Minimally invasive techniques have become standard for many abdominal procedures, but open-access laparotomy remains common, and sometimes essential, especially in complex tumor debulking procedures, trauma and emergency surgery and child delivery through cesarean Section. [1]. Laparotomy is associated with a high risk for abdominal wall complications such as wound infection (17–29% [2]) wound dehiscence (2,2–5,6% [3]) and incisional hernia formation (21%-31,8% [4]).

One fourth of patients undergoing incisional hernia repair will need a re-recurrence hernia repair and the risk of persistent pain after repair is 9–19% at one year follow-up [5, 6]. Patients with incisional hernia have reduced quality of life, suffer from impaired physical function including limitations to exercise and sex life [7]. The annual US cost for incisional hernia repair alone increased from $3.2 billion 2006 to a staggering $7,3 billion in 2011 and the individual and societal burden of abdominal wall complications is heavy, making identification and implementation of preventive measures a priority [8, 9].

Other abdominal wall complications occur because of impaired wound healing. How well a wound will heal is affected not only by patient factors such as comorbidities and wound contamination, but also surgeon attentive factors such as tissue handling and suturing tension [10]. The risk of such complications including wound dehiscence, wound infection and incisional hernia formation are reduced when a slowly resorbable suture is used, and the suture line is continuous with a suture-length-to-wound-length ratio (SL/WL ratio) of at least 4 and is deployed with small-bites [2, 11–13].

The abdominal wall is typically closed in two layers – the fascial layer and the skin – when the intra-abdominal part of the operation is finalized. The size of a bite is the distance from the wound’s edge to the point of entry of the needle [14]. Small-bites avoid incorporation of fatty tissue and muscle into the bite. In clinical trials this is achieved by instructing the surgeon and using a smaller suture-needle that restricts bite size [11, 13]. Large-bites have a bite size > 10 mm and a step interval of 10 mm. For large-bites, a larger suture needle is used that incorporates more tissue. The length of the suture deployed in the wound should be measured and divided with the length of the wound to calculate the SL/WLratio which should be at least 4. In practice, small-bites implies fascial bites of five to eight millimeters and interval steps of five milimeters. The effectiveness of the small-bites technique has been assessed in several clinical trials, both in elective and emergency settings, and small-bites is now recommended by the Joint European and American Hernia Societies guidelines from 2022 and the World Society of Emergency Surgery guidelines from 2023 [15, 16].

Even though the clinical benefit of small-bites has been recognized in guidelines since 2015, uptake of the practice by surgeons remains uncommon. In a questionnaire-based Dutch study from 2019, less than one quarter of the responders practiced fascial bites with steps < 5 mm and only 35% preferred a suture-length to wound length ratio of 4:1 [17]. In a survey from 2019 where respondents included members of Americas Hernia Society, European Hernia Society and the International Hernia Collaboration, 19% stated they did not practice small-bites because it doesn’t apply to the patient population, 24% stated they were not familiar with the technique, and 13% stated the procedure takes too long [18]. Thus any developments that allow for the quicker and more efficient implementation of the small-bites technique could facilitate and improve uptake.

**Fig. 1:**
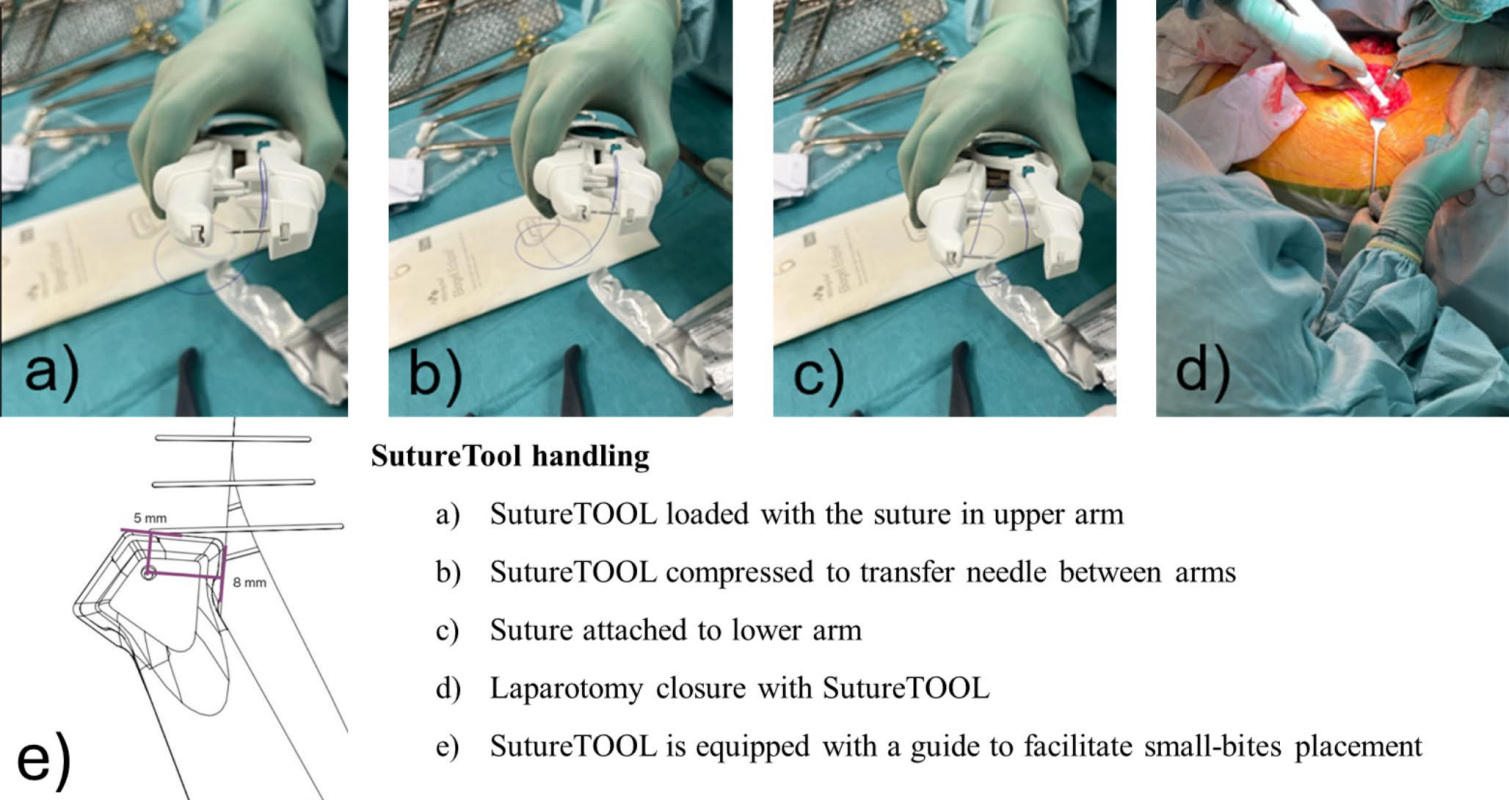
Description of SutureTool handling

To facilitate implementation of the small-bites technique a suturing device, SutureTOOL (Suturion, Lund, Sweden) has been developed for swift and standardized laparotomy closure [19]. The device was developed by the second author (GB) in collaboration with Lund University, Lund Sweden. The device is a sterile, single use, handheld, mechanical laparotomy closure device with a double pointed needle. The suture needle has a centrally attached 130 cm long polydioxanone 2/0 suture thread. SutureTOOL has a guide that facilitates small-bites stitch placement – a 5–8 mm bites size and a 5 mm step interval. SutureTOOL and device suturing is explained in Fig. 1. Small-bites are enabled by a guide that indicates the measurements of small bites and facilitates correct stitch placement. Suture time, defined as the duration from the first stitch to the final stitch in the aponeurosis, is generally longer for small-bites suturing compared to large-bites suturing. However, the SutureTOOL has been shown to reduce suture time while ensuring small-bites suturing [19, 20]. It has been evaluated in pre-clinical studies where a SL/WL ratio of 4 was achieved in 95–98% of patients and in a recently completed first-in-man clinical trial where a SL/WL of 4 was achieved in 100% of patients.

To our knowledge there are two previous publications assessing the health economic cost-effectiveness of small-bites. Millbourn et al. [21] included data from a Swedish randomized clinical trial (2001–2006) comparing postoperative complications after laparotomy closure with small-bites and large-bites on patients undergoing laparotomy for colorectal cancer. The health economic analysis was performed from a societal perspective, included direct and indirect costs, and found a cost reduction of 2,415 SEK in patients that had a midline incision with small-bites, approximately $230 in 2013. Gokani et al. [22] performed a cost-utility analysis to compare outcomes after small-bites and large-bites technique for laparotomy closure and the change in quality-adjusted life years (QALYs) with the UK public health provider National Health Service (NHS) perspective rather than a societal perspective. The study assessed the cost implications of adopting small-bites as standard practice and the impact of small-bites as standard practice on QALY measures. The analysis of the added operation time (4,6 min) cost for small-bites suturing (£92 per patient) deemed small-bites to be cost-effective provided a decrease in absolute wound infection rates by more than 15%, or absolute incisional hernia rates by more than 3.4%. While SutureTOOL facilitates small-bite laparotomy closure, the additional equipment comes with a cost and changes how the procedure is performed. The cost-effectiveness of the SutureTOOL, accounting for these changes, has not yet been explored to inform procurement decisions.

The aim of the study was to compare the economic and clinical outcomes of two laparotomy closure techniques performed on patients undergoing open abdominal surgical procedures: needle-driver suturing (manual suturing) as standard care and device-assisted suturing (SutureTOOL).

## Methods

### Study design

This was a cost-effectiveness analysis comparing the economic and clinical outcomes of device-assisted suturing as compared to those of needle-driver suturing as standard care. Thus, the study population includes patients undergoing laparotomy closure. It should be noted that all data used in this model analysis was taken from published peer-reviewed literature, and no primary data was collected for this evaluation. The analysis was performed from a healthcare perspective as decisions to procure the device will likely be made from this perspective, and it is a conservative analytical decision to forgo a societal perspective. Societal effects likely pertain to productivity loss and patient/caregiver travel costs associated with long-term impacts of complications. Needle-driver suturing is the current standard in each country context, and the evidence suggests that the SutureTOOL reduces operation time and increases the percentages of operations performed using small-bites – thus reducing complication rates. Resource use and unit costs data were sourced from four country contexts: Sweden, France, The United Kingdom, and The United States. The analysis is therefore performed separately for each country-context.

A decision tree model was developed in Microsoft Excel (version 24.04) to implement this comparison, and the analysis was performed over a time horizon of 3 years where year-one begins at the index operation. This timeframe was sufficient to capture key postoperative healthcare-perspective costs and outcomes [23], particularly those associated with incisional hernias which tend to present between one and three years following the operation. Inputs to the model were obtained through a targeted literature review and through expert opinion.

### Model structure

**Fig. 2:**
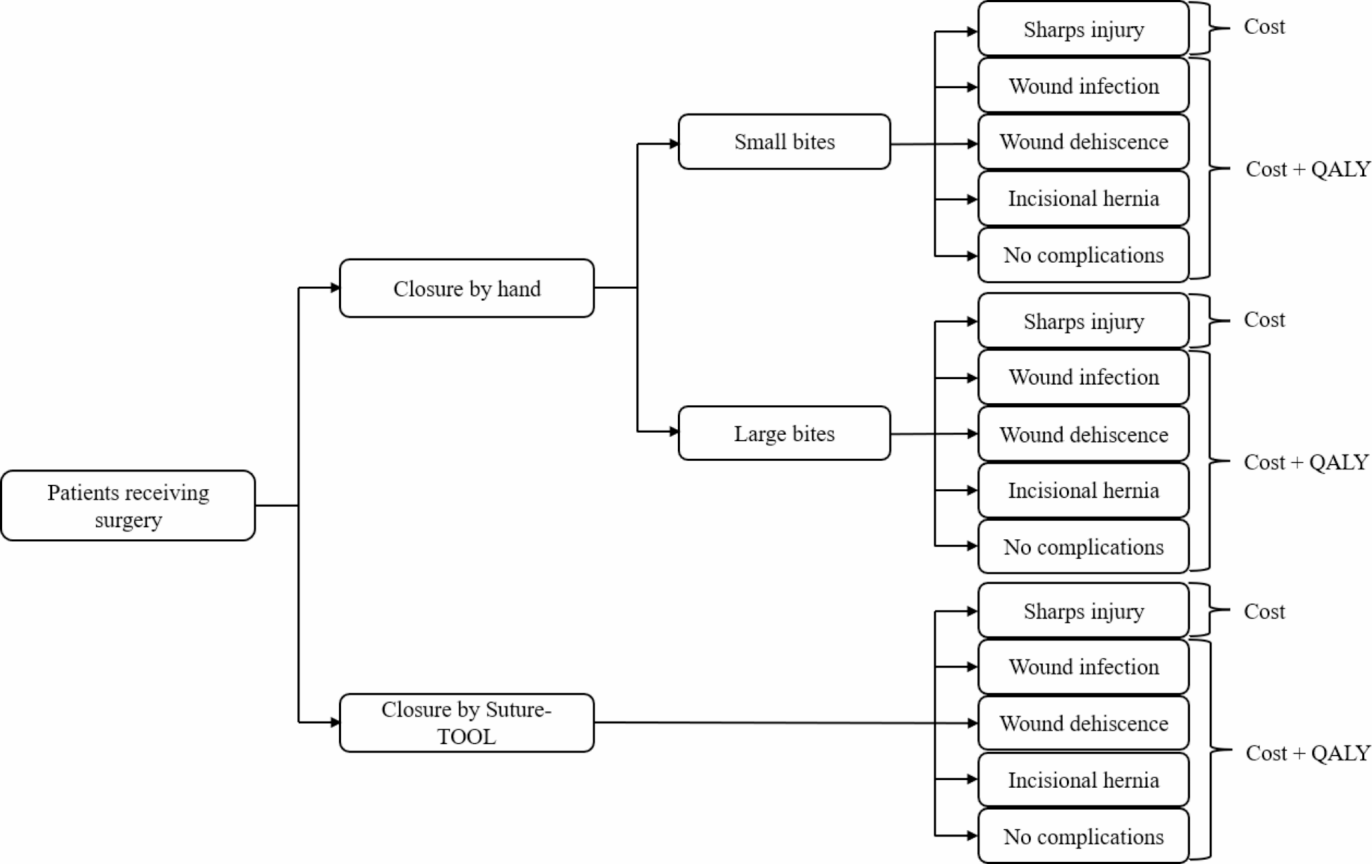
Decision tree model

A decision tree model was constructed to assess the probability and impact of complications associated with each laparotomy closure technique (Fig. 2). Surgeons’ adherence to the recommendation of small-bites suturing significantly influences complication rates, including wound infection, wound dehiscence, and incisional hernia [2, 13]. Previous experimental and clinical studies have estimated that small-bites achieved by manual needle-driver suturing can vary from 30% to 76% [20, 24, 25] while the SutureTOOL ensured 95–98% small-bites suturing [20]. In our base-case analysis, we assumed that small-bites were used in 50% of needle-driver sutures and 100% of sutures by the SutureTOOL. In a recent un-published clinical trial, 100% of the 38 laparotomy closures achieved SL/WL-ratio of at least 4 with the SutureTOOL. Costs and outcomes were calculated for each suturing technique.

The probabilities of surgical complications were estimated from annual event risks reported in the published literature (Table 1). Sharps injury is an intra-operative complication, and wound infection and dehiscence occur within the first year following the operation – most within the first six weeks. Most incisional hernias manifest within the first three years after surgery, and studies indicate that the risk for incisional hernia is generally distributed over three years: 40% in the first year, another 40% in the second year, and the remaining 20% in the third year [23]. Approximately 65.3% of hernia do not require surgical repair [26].

The frequencies of the three high-cost surgical complications – wound infection, wound dehiscence, and incisional hernia – have substantial implications for model results. While previous studies are somewhat consistent in the general range of estimates of event frequencies, the difference in event rates between small and large bites is somewhat inconsistent (see Supplementary Materials 2). We therefore identified what we see as the most appropriate event rates for the analysis, and include best- and worst-case scenario analyses within the Supplementary Materials 2. Deerenberg et al. [11] was a randomized controlled trial (RCT) published in the Lancet in 2015. The target outcome of the trial was incisional hernia, and the article was thus used to source IH rates for small and large bites in the base-case analysis.

We chose not to use the STITCH study [11] results for wound infection in the base case of our study as we regard the results from Millbourn et al. [13] as a more appropriate choice. In the STITCH study, wound infection rates were considered secondary outcomes, whereas the Millbourn et al. [13] study was powered specifically to assess wound infection, which was among the trial’s primary outcomes. Milbourn also had the largest sample size and was conducted in Sweden, a country included in our analysis.

Wound dehiscence rates were obtained fromAlbertsmeier et al. [27], a recent RCT conducted in 2022. The results were not statistically significant, but the p-value was 0.0513, just outside of statistical significance. Wound dehiscence was the main outcome of the study.

**Table 1:** Suture time and complication risks by suturing technique

Parameters	Values	Sources
Suture time (min)		
Small-bites	14	[11]
Large-bites	10	[11]
SutureTOOL	6.5	Unpublished clinical trial
Sharps injury (%)		
Small-bites	17.0	[28]
Large-bites	17.0	[28]
SutureTOOL	0.0	[19]
Wound infection (%)		
Small-bites	5.0	[13]
Large-bites	10.0	[13]
SutureTOOL	5.0	Assumed same as small bites
Wound dehiscence (%)		
Small-bites	1.0	[27]
Large-bites	5.0	[27]
SutureTOOL	1.0	Assumed same as small bites
Incisional hernia (%)		
Small-bites	32.5	Calculated from Deerenberg [29] Deerenberg et al. [11] and Brandl et al. [23], See Supplementary Materials 1
Large-bites	52.5	
SutureTOOL	32.5	Assumed same as small-bites
% IH presenting in		
1st year	40.0	[23]
2nd year	40.0	[23]
3rd year	20.0	[23]
% IH non-operative	65.3	[26]

### Efficacy

The main outcomes used to assess the clinical efficacy of each surgical intervention were Life years (LY) and quality-adjusted life years (QALY). The QALY serves as a comprehensive metric, encompassing both the quantity and quality of life experienced by individuals. This composite measure integrates health-related quality of life and life expectancy, enabling a holistic evaluation of treatment efficacy. A health weight, also called a utility weight, reflects an individual’s quality of life and is measured on a scale from 0 (indicating deceased status) to 1 (indicating perfect health). Utility values, alongside their respective duration and mortality risk of surgical complications, were used in the calculation of LYs and QALYs in this study, and are presented in Table 2.

**Table 2:** Utility values and mortality risk of surgical complications

Parameters	Values	Sources
Utility		
Wound infection	0.56	[30]
Wound dehiscence	0.53	[30]
Incisional hernia	0.75	[31]
Duration of health state		
Wound infection	0.038	Estimate of co-author clinician
Wound dehiscence	0.076	Estimate of co-author clinician
Incisional hernia	0.5	Assumption
Mortality risk (%)		
Wound infection	2.9	[32]
Wound dehiscence	16.0	[33]
Incisional hernia	0.2	[34]

### Costing

This study included direct costs incurred from a healthcare system perspective, including suturing materials, operating theater expenses, and costs related to surgical complications. Manual suturing was assumed to require two sutures per abdominal wound operation on average. For device-assisted suturing (SutureTOOL), the cost included both the device itself and the sutures. Operating theater costs were calculated on a per-minute basis, while costs associated with surgical complications were estimated per event. All costs except those associated with incisional hernia were incurred during the operation or within the first year after operation. Costs related to incisional hernia were spread over a 3-year period, reflecting the accumulated risks over time. Unit costs, along with their respective sources for each study country, are presented in Table 3.

**Table 3:** Unit costs

Parameters	Sweden (SEK)		UK (£)		US ($)		France (€)	
	Values^#^	Sources	Values^#^	Sources	Values^#^	Sources	Values^#^	Sources
Manual suturing*	128.00	Assumption	11.40	Assumption	12.00	Assumption	11.40	Assumption
SutureTOOL^†^	2864.00	Market Price	266.70	Market Price	$381	Market Price	290.70	Market Price
Operating theater cost (per min)	303.18	[21]	21.03	[22]	47.02	[35]	10.78	[36]
Sharps injury	2929.41	[37]	435.45	[38]	2604.92	[39]	262.41	[40]
Wound infection	80005.95	[41]	4891.58	[42]	23501.37	[43]	3767.73	[44]
Wound dehiscence‡	180820.01	[33]	11055.38	[45]	53115.02	Assumption	8515.37	Assumption
Incisional hernia	59044.69	[21]	1881.78	[22]	30712.05	[46]	6500.74	[47]

*Estimated from the cost of two sutures in each country context

†Sutures are included in the cost of device – 2 sutures according to a completed, yet unpublished, clinical trial

‡ The ratio of dehiscence-to-wound-infection-costs in US was applied to the wound infection costs in all other countries

^#^All costs have been updated to 2024 prices using country-specific HICPs for healthcare

### Base case analysis

In the base-case scenario, model inputs were set to best mirror a typical patient cohort and relevant clinical settings. Patients were assumed to have an average baseline age of 70 years, consistent with the demographic commonly undergoing abdominal surgeries [4]. A discount rate of 3% was applied to emphasize direct cost comparisons.

### Sensitivity analysis

To assess the robustness of the model and identify key model drivers, deterministic sensitivity analyses were performed by systematically adjusting key parameters by ± 50%. This analysis involved altering one parameter at a time while holding all others constant to assess the impact of each variable on model results.

### Model validation

To bolster the reliability of the decision tree model, a comprehensive validation process was conducted. Internal validation involved rigorously checking the model’s calculations and logic, model walk throughs, extreme value tests, and sensitivity analyses testing the stability of outcomes against variations in key parameters. The model underwent face validity through expert review by a panel of healthcare economists, and surgeons, who assessed the assumptions, data sources, and methodologies used. Calibration was performed by adjusting parameters to match observed data from Sweden, UK, US, and France.

## Results

### Base case analysis

Device-assisted suturing (SutureTOOL) showed a reduction in operation-theatre time by 5.5 min per patient as compared to manual suturing (Table 4). The SutureTOOL also eliminated the risk of sharps injury during surgeries. In terms of post-operative outcomes, the use of the SutureTOOL was associated with a reduction, in absolute terms, in several complications: wound infections by 2.39%, wound dehiscence by 1.65%, and incisional hernia by 16.11%. Consequently, device-assisted suturing generated an estimated savings of 0.01 life years or 0.04 quality-adjusted life years (QALYs) per patient.

The reduction in suture time resulted in lower operational theater costs and decreased cost for surgical complications and their associated expenses. The total cost per patient using the SutureTOOL was lower than that of manual suturing in all study countries with savings of SEK 6,744 in Sweden, £310 in the UK, $2,996 in the US, and €321 in France. Additionally, the incremental cost per QALY gained was negative in all of these countries, indicating lower costs for each QALY gained. This indicates that the SutureTOOL is cost-effective when compared to manual suturing.

**Table 4:** Outcomes of abdominal operation by suture techniques

Results per patient	Manual suturing	SutureTOOL	Difference
Suture time (min) per patient	12	6.5	-5.5
Incidence (%)			
Sharps injury	17.0	0.0	-17.0
Wound infection	7.18	4.79	-2.39
Wound dehiscence	2.48	0.83	-1.65
Incisional hernia	31.06	14.95	-16.11
Life-Years	2.81	2.82	0.01
QALYs	2.46	2.50	0.04
Cost per patient			
Sweden (SEK)	20,724	13,980	-6,744
UK (£)	1,159	849	-310
US ($)	7,194	4,198	-2,996
France (€)	1,353	1,032	-321
Incremental cost per QALY gained		Dominant*	

*The SutureTOOL is dominant over manual suturing in all study countries

#### Sweden

**Fig. 3:**
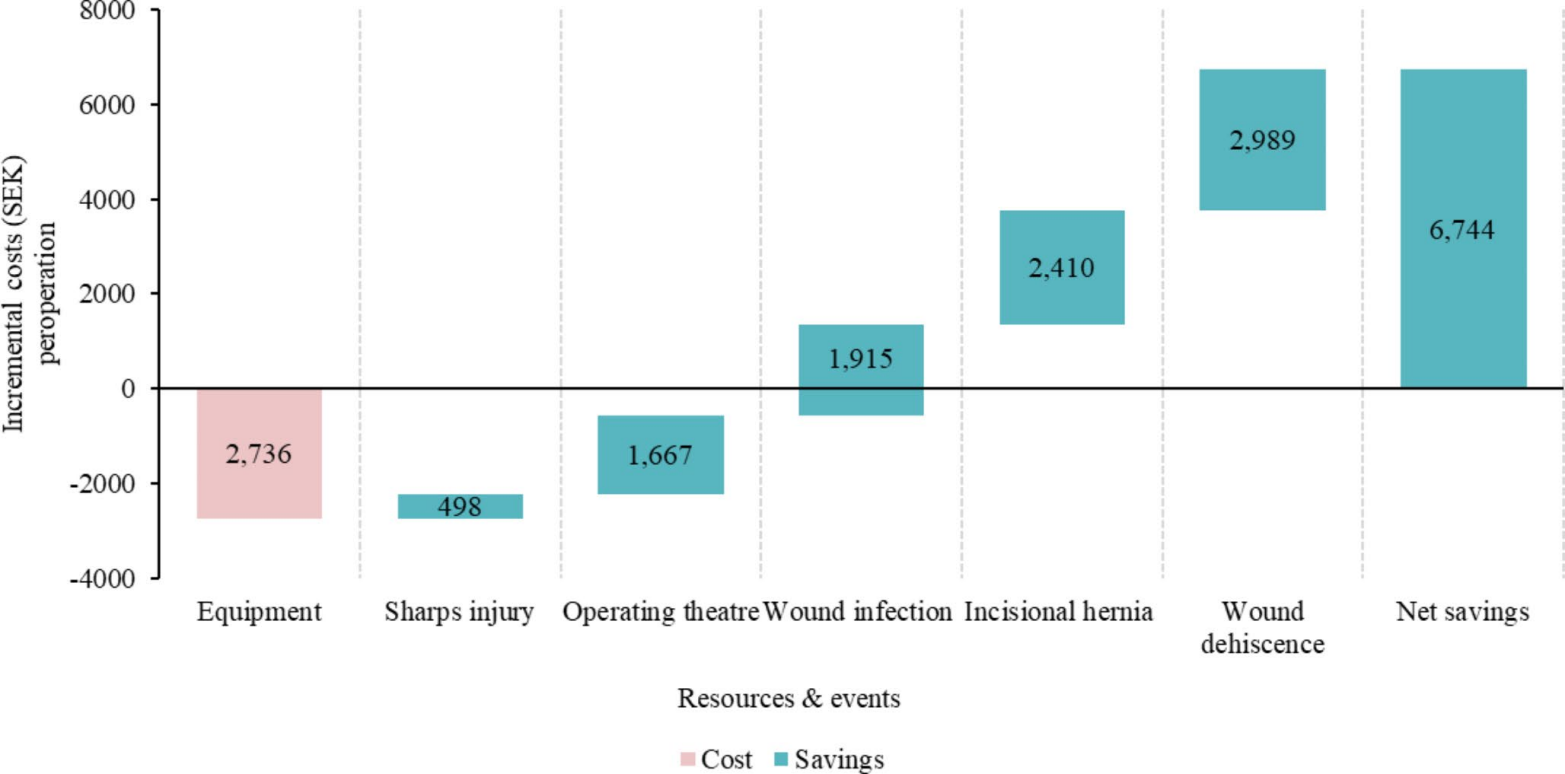
Incremental costs of using the SutureTOOL laparotomy closures compared to manual suturing in Sweden

In Sweden, the use of the SutureTOOL for laparotomy closures resulted in potential savings of SEK 6,744 per operation (Fig. 3). The device significantly reduced operating time, saving an estimated SEK 1,667 per operation. It also minimized the risk of sharps injuries to surgical providers, preventing potential costs of approximately SEK 498 per operation. Additionally, post-operative benefits included a reduced likelihood of wound infections (SEK 1,915), wound dehiscence (SEK 2,989), and incisional hernias (SEK 2,410).

#### United Kingdom

**Fig. 4:**
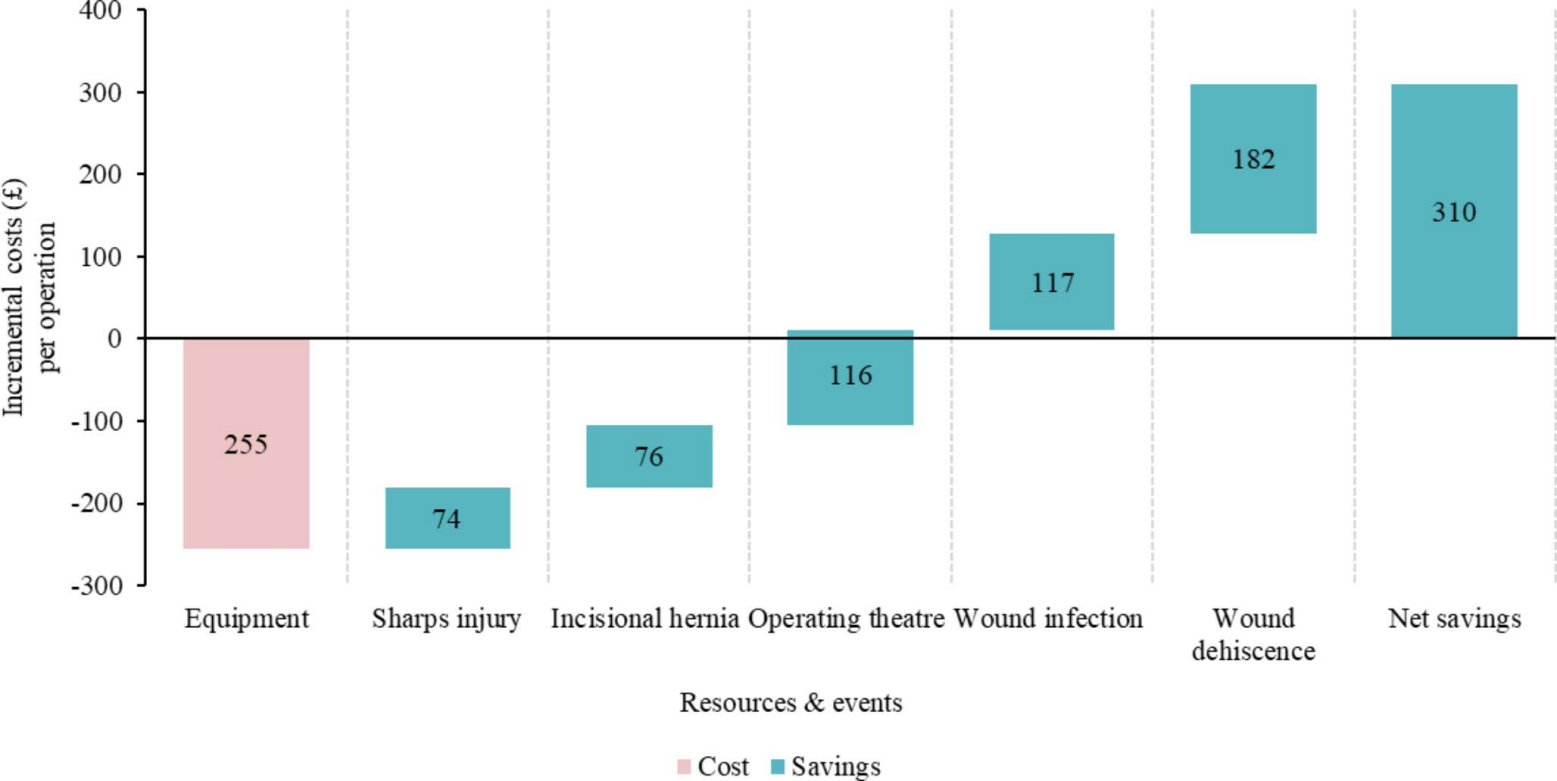
Incremental costs of using the SutureTOOL in laparotomy closures compared to manual suturing in the UK

In the UK, the implementation of the SutureTOOL resulted in potential cost savings of £310 per operation (Fig. 4). The device substantially reduced operating time, saving an estimated £116 per operation. It also decreased the risk of sharps injuries to surgical providers, avoiding potential costs of approximately £74 per operation. Additionally, post-operative benefits included a lower likelihood of wound infections (£117), wound dehiscence (£182), and incisional hernias (£76).

#### United States

**Fig. 5:**
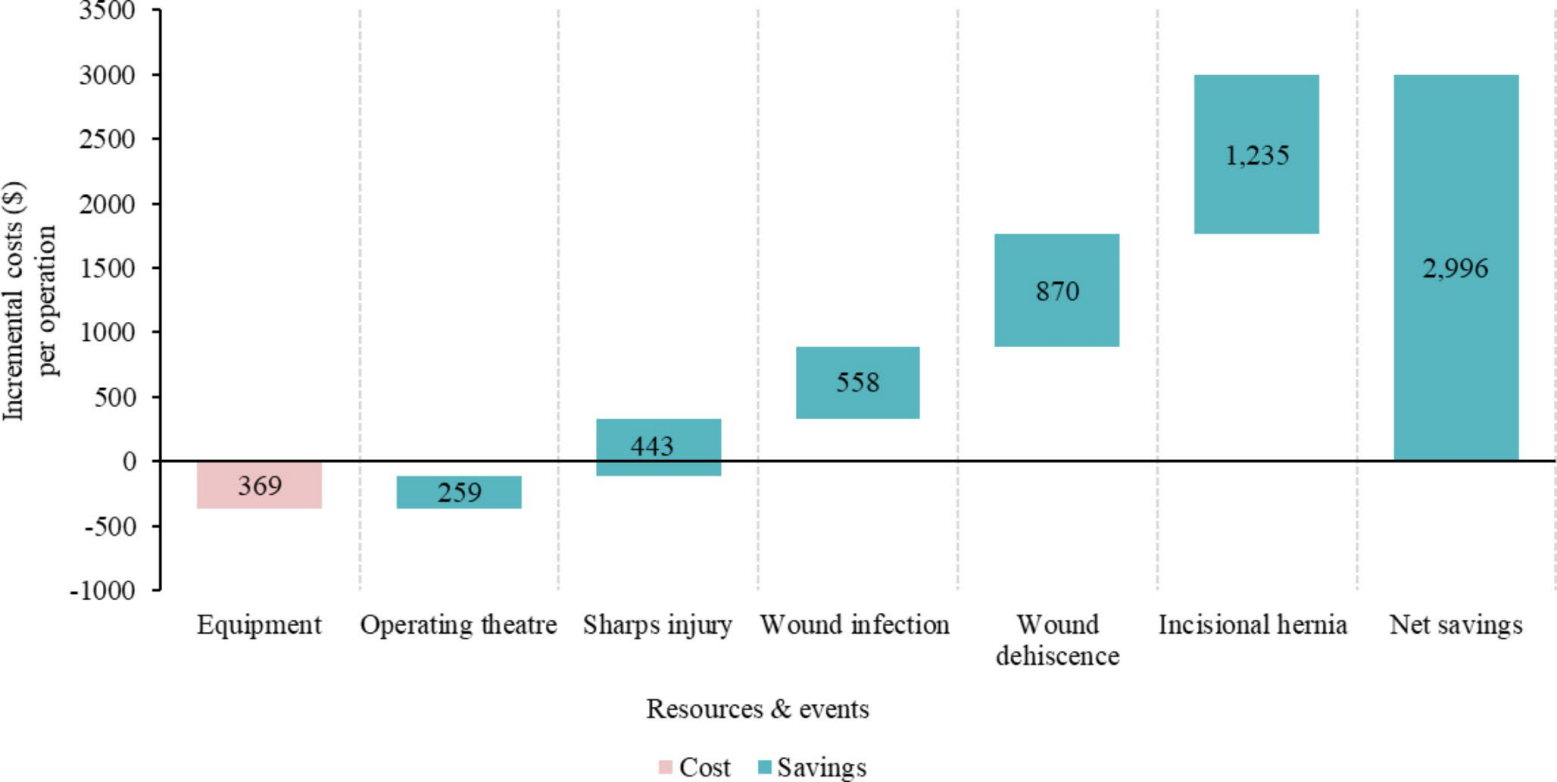
Incremental costs of using the SutureTOOL in laparotomy closures compared to manual suturing in the US

In the US, the introduction of the SutureTOOL resulted in potential cost savings of $2,996 per operation (Fig. 5). Intra-operatively, the device notably decreased operating time, saving $259 per operation. It also reduced the risk of sharps injuries to surgical providers, avoiding potential costs of approximately $443 per operation. Additionally, post-operative benefits included a decreased likelihood of wound infections ($558), wound dehiscence ($870), and incisional hernias ($1,235).

#### France

**Fig. 6:**
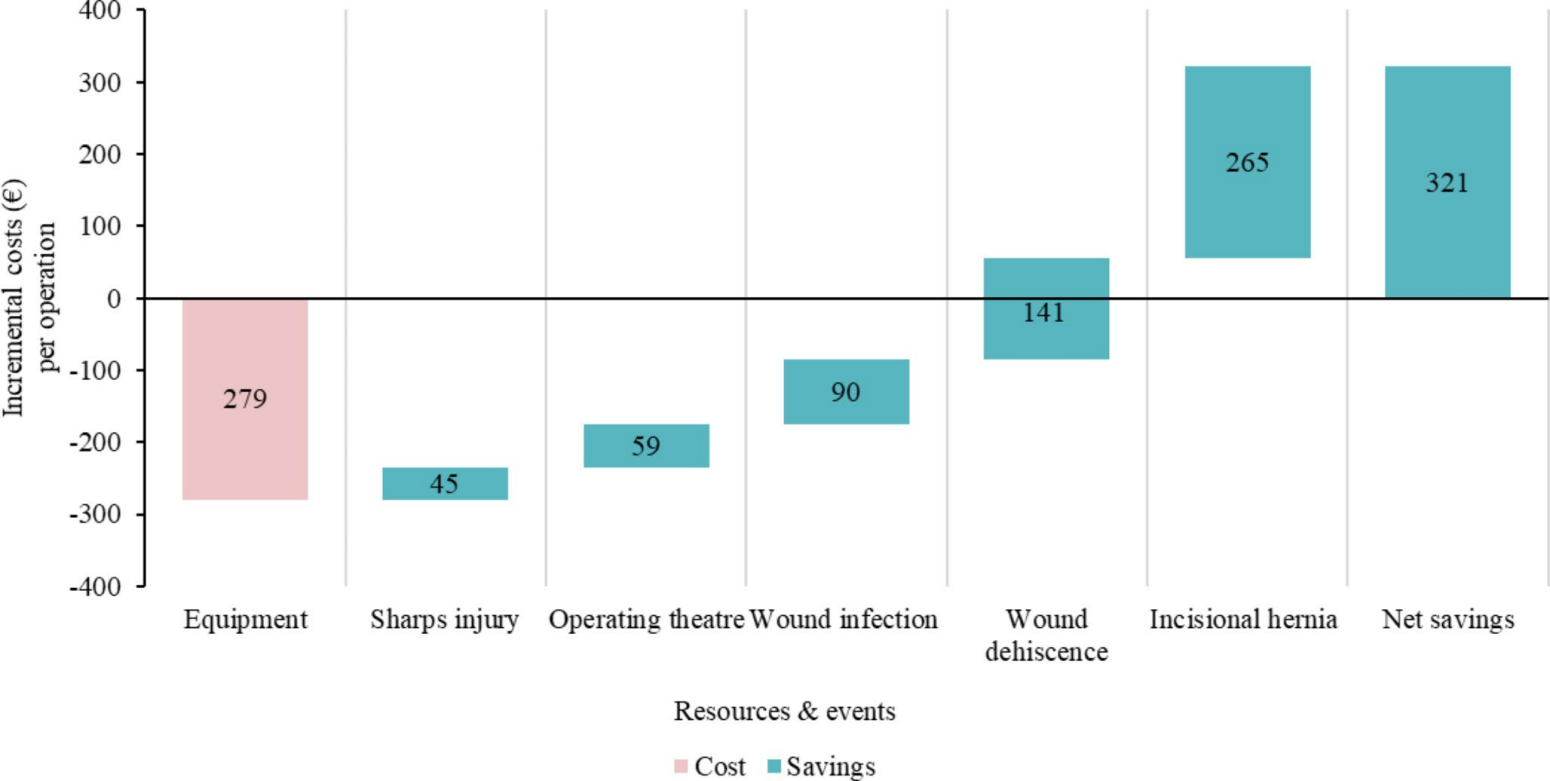
Incremental costs of using the SutureTOOL in laparotomy closures compared to manual suturing in France

In France, the integration of the SutureTOOL demonstrated promising potential cost savings of €321 per operation (Fig. 6). The device decreased operating time, saving €59 per operation. It also lowered the risk of sharps injuries to surgical providers, avoiding potential costs of approximately €45 per operation. Post-operative benefits included reduced likelihoods of wound infections (€90), wound dehiscence (€141), and incisional hernias (€265).

### Sensitivity analysis

**Fig. 7:**
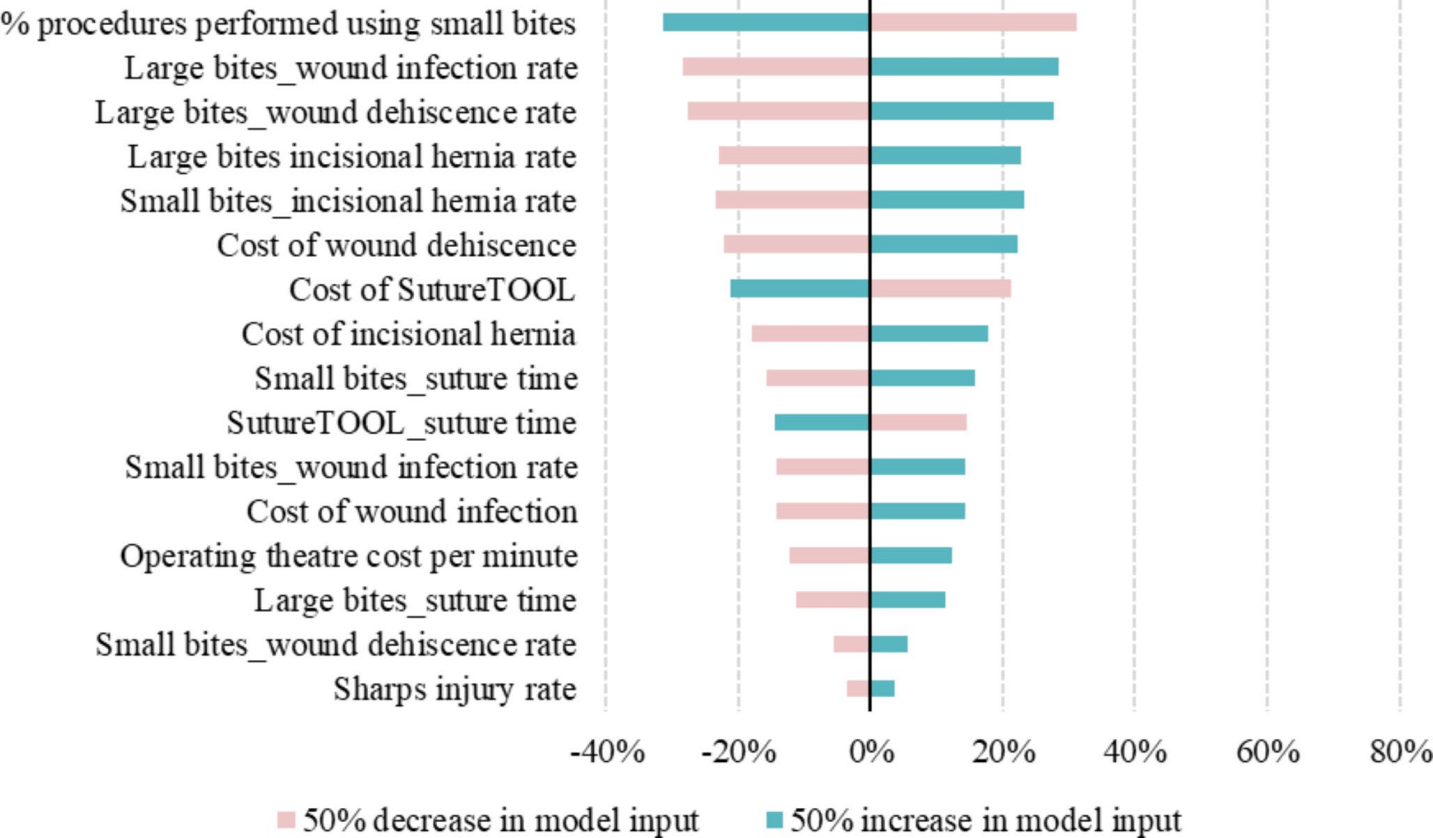
Tornado diagram presenting the deterministic sensitivity analyses

Sensitivity analyses identified key model drivers, including the proportion of manual needle-driver suturing procedures utilizing the small-bites technique, as well as the risks of wound infection, dehiscence, and incisional hernia (Fig. 7). The proportion of small-bites procedures directly influences laparotomy closure integrity and associated complication rates. Additionally, the analysis is sensitive to post-operative complication risks.

## Discussion

This study indicates that from a healthcare perspective device-assisted suturing, with the SutureTOOL, is cost-effective as compared to manual needle-driver suturing. The results indicate that using the SutureTOOL for laparotomy closures can reduce operation theatre time, thereby improving operational efficiency and lowering costs associated with theatre use. Additionally, the intervention led to a reduction in complication rates, highlighting its potential to enhance patient safety and reduce healthcare expenses. Through ensuring the use of small bites, the use of the SutureTOOL reduced the risk of sharps injuries to surgical providers, mitigating related costs, and lowered the incidence of surgical complications such as wound infections, wound dehiscence, and incisional hernia, positively impacting patient recovery and healthcare resource utilization. Finally, the analysis indicates that the SutureTOOL improves survival. In contexts where at least half of patients are operated on with large bites, for every thousand patients operated on with the SutureTOOL, an estimated three lives are saved, along with the acquisition of nine quality-adjusted life years (QALYs). The results indicate substantial cost-saving potential across multiple countries. SutureTOOL is estimated to reduce costs associated with laparotomy closure by 42% in the US, 33% in Sweden, 27% in the UK, and 24% in France. Direct costs (operating theatre costs) associated with laparotomy closure are notably reduced, because of reductions in operating time, in all countries except France where the cost of the device is higher than estimated operating theatre costs. Savings grow further when considering the impact of avoided complications, and further still when including avoided incisional hernias beyond the first year. In Sweden, total savings increase substantially when incisional hernias occurring in the second and third years are included. In all country contexts, cost savings from incisional hernias are substantially reduced because 65% of patients are estimated to not need re-operation, and savings would be higher in contexts with higher re-operation rates. Additionally, costs of complications associated with incisional hernia were not included to avoid double counting, as these costs were included with incidents of wound infection and dehiscence.

The unit cost of wound infection emerges as the highest among considered surgical complications across all studied countries, except France where it is the second highest after incisional hernia. Notably, the unit cost of incisional hernia exceeds that of wound dehiscence in Sweden and France, while the reverse is true in the US and UK. This variation in cost distribution leads to slight differences in overall net savings across different countries. These nuanced differences underscore the well-established importance of contextually valid event rates and unit-cost data if the analysis is to effectively inform decision making [48].

Key model drivers included the percentage of procedures performed using small-bites, wound infection rates, dehiscence rates, and incisional hernia rates. These parameters significantly influence the model’s results and warrant careful consideration in clinical decision-making. These key parameters may change as model inputs are tailored to other countries or provider settings. Regardless, these inputs reflect critical aspects of surgical practice and technique and underscore the importance of adopting evidence-based approaches to laparotomy closure.

This study was performed under a set of methodological limitations. Variations in healthcare practices and costs between countries and providers will affect results, and large variations could lead to changes in key model drivers. In France for example, the authors’ clinical contacts indicated that the per-minute operating room cost is too low, however the most relevant literature available indicated a cost of 10.78 Euros [36]. It is crucial that decision makers carefully consider their own clinical context and adjust the analytical approach accordingly. The analysis assumed that 50% of wound closures are performed with small-bites, which was a key model driver. The sensitivity analysis (Fig. 7) suggests that an increase in the percentage of small-bites in provider settings is associated with less cost-effectiveness when implementing the suture tool. A Dutch study reported that only 24% of clinicians perform interval steps of 5 mm or less [17]. If this rate reflects international practice, our analysis indicates our results are conservative, particularly with respect to savings associated with avoided surgical complications. No other studies were found that reported small-bites rates. Clinical settings with higher proportions of small-bites will see less savings in terms of avoided complications and more gains associated with time savings. The opposite would be true when large-bites are the dominant practice, and, importantly, these changes in savings are sensitive to variation across clinical contexts with respect to surgical complication rates and suture time. The time improvements associated with the use of SutureTOOL will also vary across contexts depending on surgical methods, the experience of clinicians, and unit costs of resources. Another key assumption included the limited time horizon and subsequent exclusion of long-term complications. As a result, the actual survival benefits could exceed the reported values, further emphasizing the potential positive impact on patient outcomes when the SutureTOOL is adopted for use. Finally, at the core of this analysis is the SutureTOOL’s ability to effectively produce SL/WL of at least 4 with small-bites suturing, reduce suture time and protect users from sharps injury. Sharps injuries often occur at the end of long and sometimes strenuous procedures, and the risk increases with 22% per hour as an operation proceeds [49]. Suturing is the most common intraoperative task where sharps injury occurs and laparotomy closure at the end of the operation where risk of injury is high [50–52]. Thus, laparotomy closure is responsible for the majority of glove punctures [52]. One feature of the SutureTOOL is that the needle track does not interfere with the user’s fingers. Glove puncture assessment has been an endpoint in SutureTOOL studies, and no punctures were recorded among 45 laparotomy closures [19].

Evidence indicates that the SutureTOOL ensures a SL/WL of at least 4 and can help surgeons adhere to guidelines for laparotomy closure and thus have the potential of reducing abdominal-wall complication rates and operating time. Prior to procuring the SutureTOOL, providers should be careful to ensure that these effects carry over into their clinical operations, and to consider the financial impact of operating time reductions according to their own per-minute operating theater costs. The SutureTOOL has been evaluated in two pre-clinical trials and one pre-market clinical trial. Within these study contexts, participants received minimal training with the SutureTOOL system. Performance could possibly be further enhanced when the practice is well-established in a broader clinical setting [19, 20]. These findings provide valuable insights into the potential benefits of adopting the SutureTOOL in clinical practice, supporting its integration as a valuable medical technology for enhancing surgical outcomes and patient care.

## Conclusion

The results of this cost-effectiveness analysis suggest that the SutureTOOL is a cost-effective intervention largely due to the substantial savings generated through reduced operation time and abdominal wall-related complications. Hospitals considering this analysis as a basis for decision making should be careful to test the device within their clinical context to ensure (1) that reductions in operation time are replicable within their clinical context, and (2) that the device consistently delivers a SL/WL ratio of at least 4 with small-bites sutures for laparotomy closure among their providers. Consistent achievement of a SL/WL ratio of at least 4 with small-bites has been shown to reduce complication rates, and the costs savings of avoided events together with those of reductions in operating room time can improve the quality of care for patients undergoing laparotomy closure.

## Electronic supplementary material

Below is the link to the electronic supplementary material.

Supplementary Material 1

Supplementary Material 2
