# Supplementary material for: A literature-based cost-effectiveness analysis of device-assisted suturing versus needle-driven suturing during laparotomy closure
Source: Hernia. 2025 Jan 23;29(1):77. doi: 10.1007/s10029-025-03266-2 (PMC11759271; doi:10.1007/s10029-025-03266-2)
Supplement: Supplementary file 1 — Supplementary Material 1 [file 10029_2025_3266_MOESM1_ESM.pdf]

Estimated percentage of patients who experience hernia

Patients experiencing incisional hernias is year 1:
(Deerenberg et al., 2015)

Small bites: 13%
Large bites: 21%

Percentage of incisional hernias ocurring in years 1, 2, and 3 following surgery:
(Brandl et al., 2014)

Year 1: 40%
Year 2: 40%
Year 3: 20%

Estimated percentage of patients experiencing hernia in the model:

Small bites: 32.5%
Large bites: 52.5%

$$Small\ bites\ IH\ Rate = \left(\frac{13\%}{40\%}\right) = 32.5\%$$

$$Large\ bites\ IH\ Rate = \left(\frac{21\%}{40\%}\right) = 52.5\%$$

Formulas explained

If 21% of patients experience a hernia in the 1st year following wound closure using small bites, and 40% of all hernias occur in the 1st year, then we can estimate that 32.5% percent of patients experience a hernia (at any time) following wound closure using small bites.

- Brandl, A., Laimer, E., Perathoner, A., Zitt, M., Pratschke, J., & Kafka-Ritsch, R. (2014). Incisional hernia rate after open abdomen treatment with negative pressure and delayed primary fascia closure. *Hernia*, *18*(1), 105-111. https://doi.org/10.1007/s10029-013-1064-0
- Deerenberg, E. B., Harlaar, J. J., Steyerberg, E. W., Lont, H. E., van Doorn, H. C., Heisterkamp, J., Wijnhoven, B. P., Schouten, W. R., Cense, H. A., Stockmann, H. B., Berends, F. J., Dijkhuizen, F. P. H., Dwarkasing, R. S., Jairam, A. P., van Ramshorst, G. H., Kleinrensink, G. J., Jeekel, J., & Lange, J. F. (2015). Small bites versus large bites for closure of abdominal midline incisions (STITCH): a double-blind, multicentre, randomised controlled trial. *Lancet*, *386*(10000), 1254-1260. https://doi.org/10.1016/s0140-6736(15)60459-7
