# Supplementary material for: A literature-based cost-effectiveness analysis of device-assisted suturing versus needle-driven suturing during laparotomy closure
Source: Hernia. 2025 Jan 23;29(1):77. doi: 10.1007/s10029-025-03266-2 (PMC11759271; doi:10.1007/s10029-025-03266-2)
Supplement: Supplementary file 2 — Supplementary Material 2 [file 10029_2025_3266_MOESM2_ESM.docx]

# Supplementary Material

Reported surgical complication frequencies and the duration of suturing time varied somewhat across the published literature. Each of these inputs are key model drivers and decided to present a best- and worst-case scenarios of the output of the analysis, only pulling data from randomized controlled trails. Table 1 presents the values of these key inputs from the identified RCTs in the literature.

Table 1: Reported per-wound closure complication rates and operation times from RCTs

|  | Small bites | Large bites | Article |
| --- | --- | --- | --- |
| Wound infection | 0.21 | 0.24 | Deerenberg, Harlaar (1) |
|  | 0.04 | 0.06 | Albertsmeier, Hofmann (2) |
|  | 0.05 | 0.10 | Millbourn, Cengiz (3)* |
| Wound Dehiscence | 0.01 | 0.01 | Deerenberg, Harlaar (1) |
|  | 0.01 | 0.05 | Albertsmeier, Hofmann (2)^⸸^ |
|  | 0.00 | 0.00 | Millbourn, Cengiz (3) |
| Incisional hernia | 0.13 | 0.21 | Deerenberg, Harlaar (1)* |
|  | 0.06 | 0.18 | Millbourn, Cengiz (3)* |
| Suturing time | 10 minutes | 14 minutes | Deerenberg, Harlaar (1)* |
|  | 14 minutes | 18 minutes | Millbourn, Cengiz (3)* |
|  | 9 minutes | 15 minutes | Albertsmeier, Hofmann (2)* |

*statistically significant at 0.05

^⸸^p-value of 0.0513

Based on these possible inputs for surgical complication frequencies, we performed a best-case and worst-case scenario analysis. The data selected are all from studies that we consider to be of high quality (Randomized controlled trials, large sample size and countries with similar characteristics). The worst-case scenario inputs are presented in Table 2. Where statistically significant differences between small and large bites was not detected, the inputs were set to be equal (no difference). The best-case scenario was inputs are presented in Table 3.

Table 2: Worst-case scenario

|  | Source | Small Bites | Large Bites | Difference |
| --- | --- | --- | --- | --- |
| Suture time for 16.5 cm wound | Deerenberg, Harlaar (1) | 14 | 10 | 2.50 |
| Wound infection | Deerenberg, Harlaar (1) | 0.21 | 0.21 | 0 |
| Incisional hernia | Deerenberg, Harlaar (1) | 0.13 | 0.21 | -0.08 |
| Wound dehiscence | Albertsmeier, Hofmann (2) | 0.01 | 0.01 | 0 |

Table 3: Best-case scenario

|  | Source | Small Bites | Large Bites | Difference |
| --- | --- | --- | --- | --- |
| Suture time | Millbourn, Cengiz (3) | 18 | 14 | 4 |
| Wound infection | Millbourn, Cengiz (3) | 0.05 | 0.10 | -0.05^⸸^ |
| Incisional hernia | Millbourn, Cengiz (3) | 0.06 | 0.18 | -0.12 |
| Wound dehiscence | Albertsmeier, Hofmann (2) | 0.01 | 0.05 | -0.04 |

^⸸^Not statistically significant, but p-value = 0.0513

# Worst-case scenario results


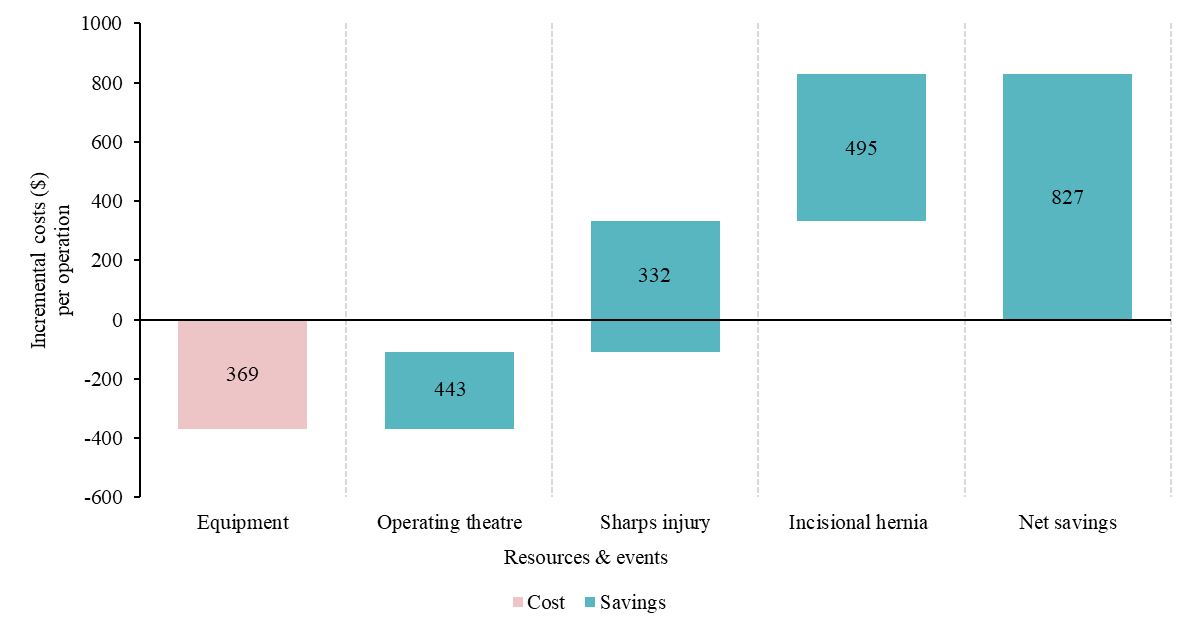


Figure 1:US worst-case results


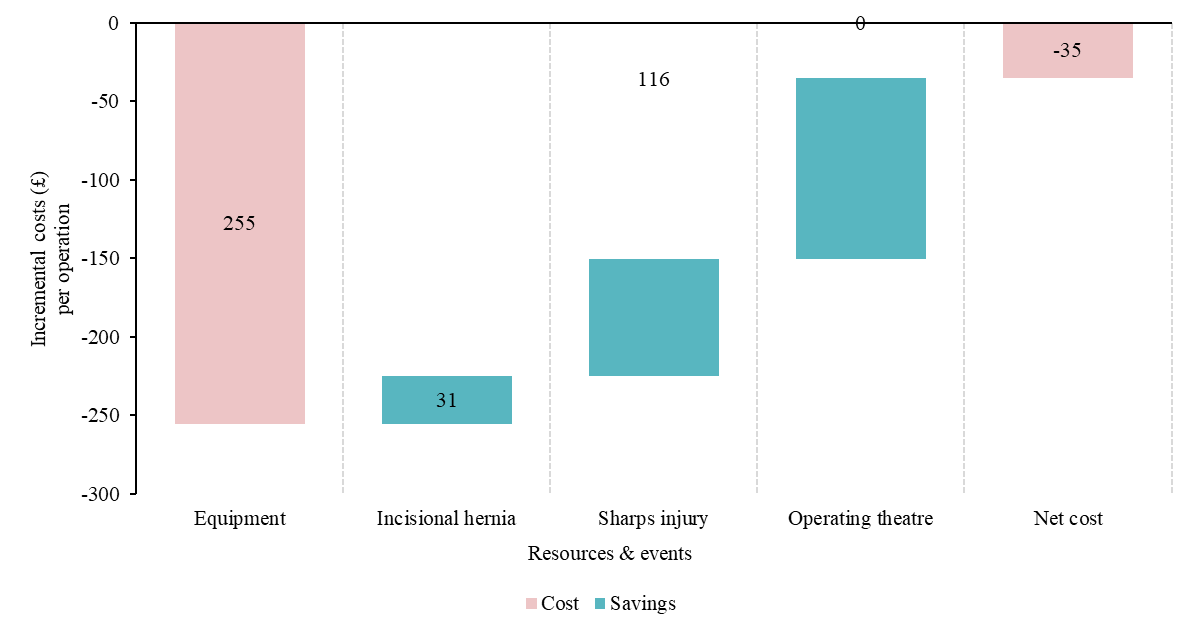


Figure 2: UK, worst-case results


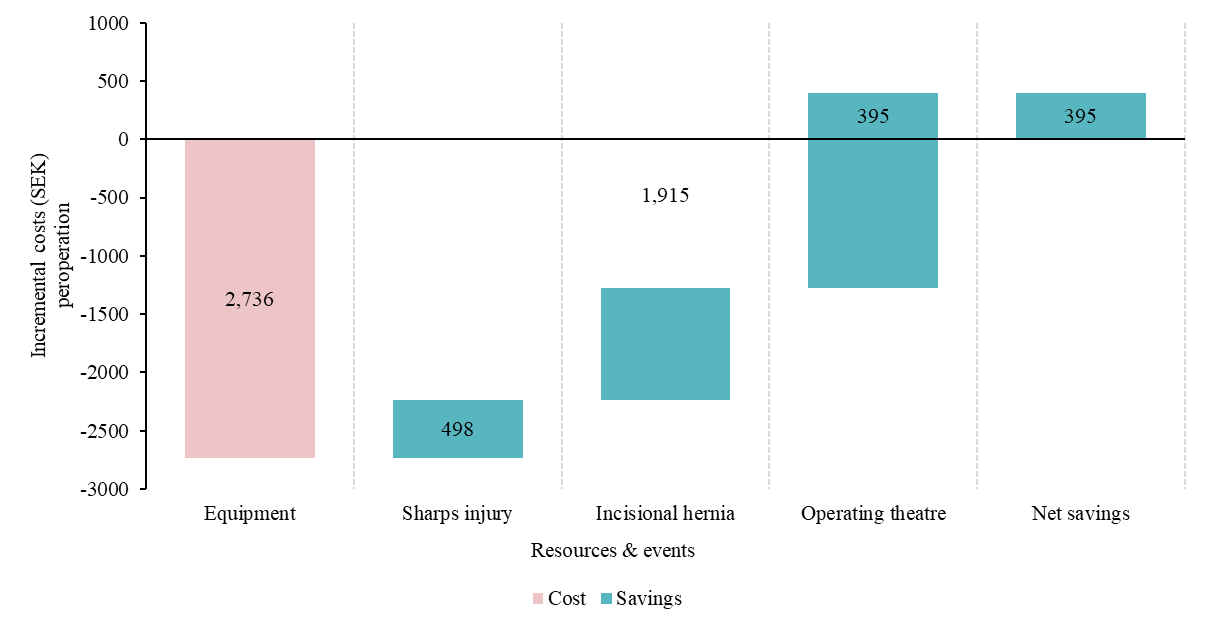


Figure 3:Sweden, worst-case results


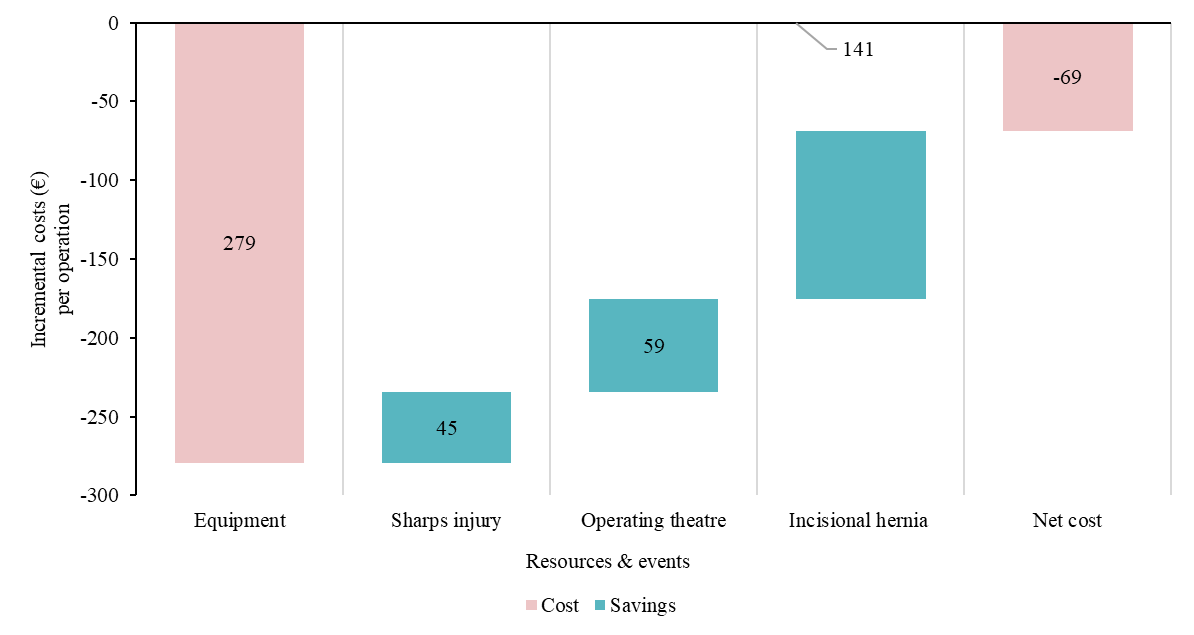


Figure 4:France, worst-case results

# Best-case scenario results


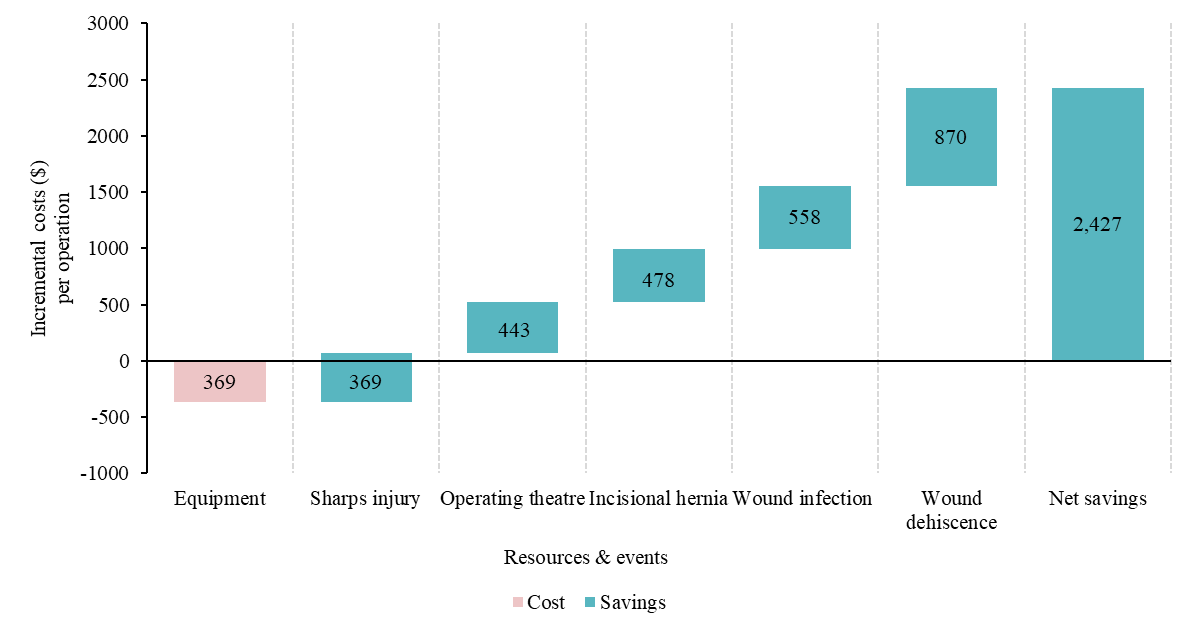


Figure 5:US, best-case results


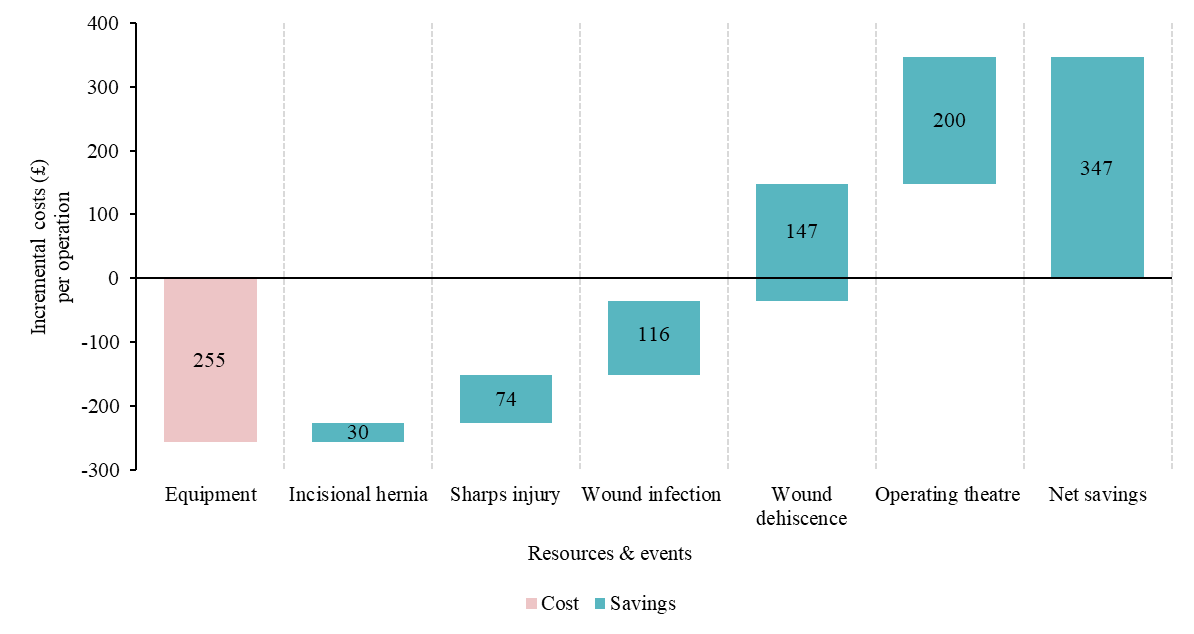


Figure 6:UK, best-case results


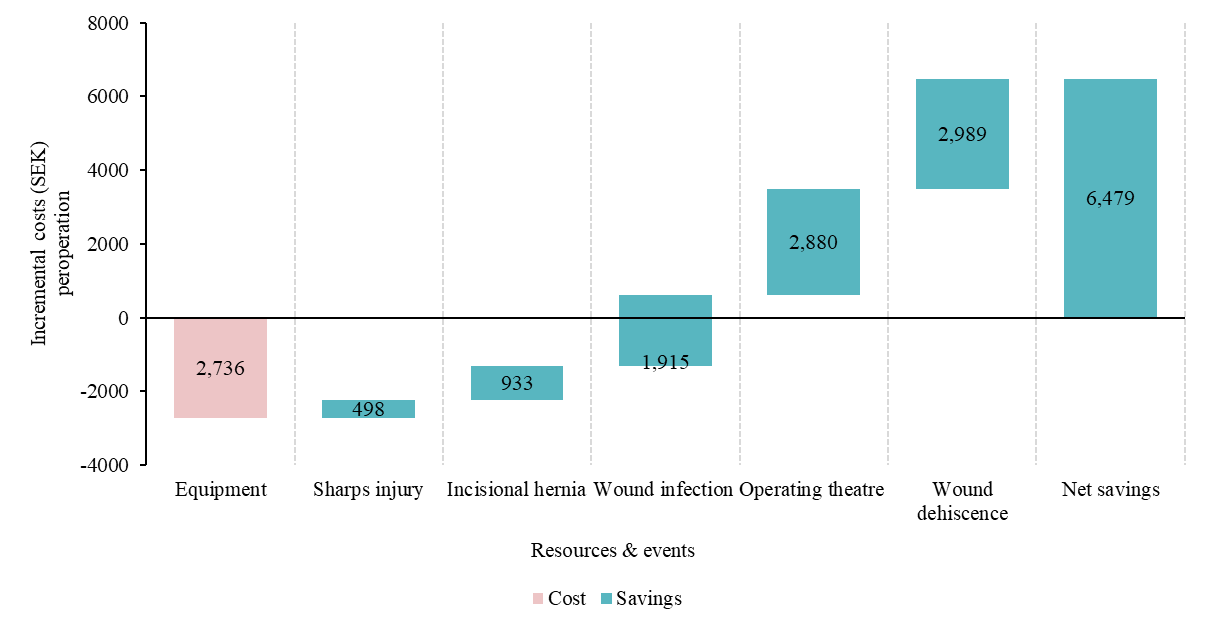


Figure 7:Sweden, best-case results


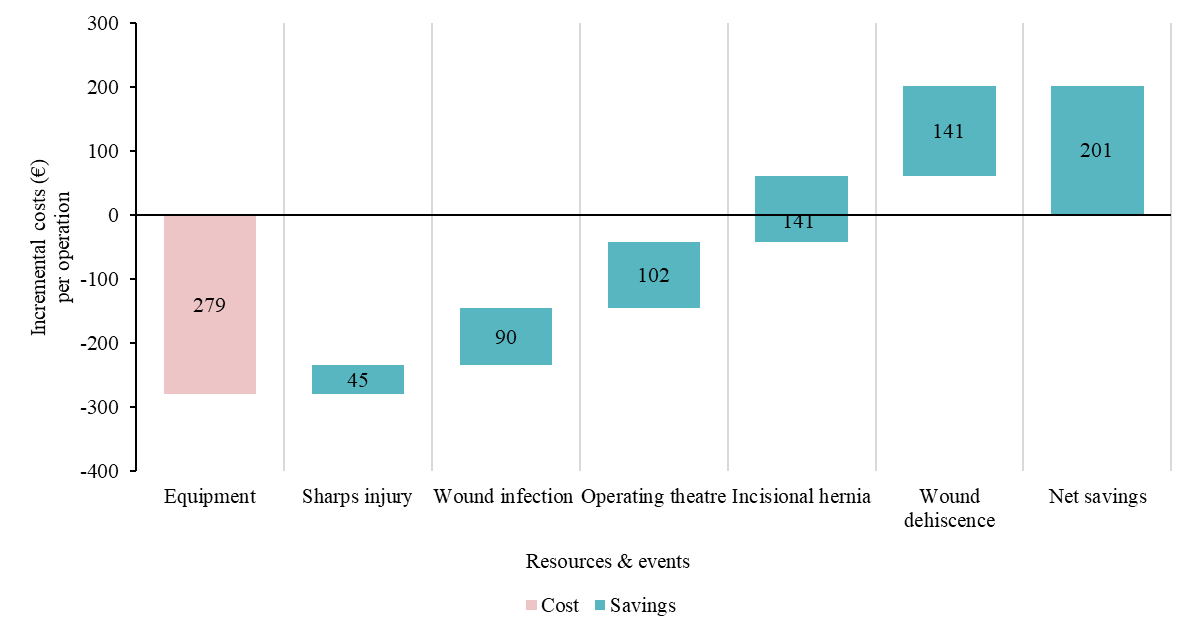


Figure 8: France, best-case results

# References

1. Deerenberg EB, Harlaar JJ, Steyerberg EW, Lont HE, van Doorn HC, Heisterkamp J, et al. Small bites versus large bites for closure of abdominal midline incisions (STITCH): a double-blind, multicentre, randomised controlled trial. Lancet. 2015;386(10000):1254-60.

2. Albertsmeier M, Hofmann A, Baumann P, Riedl S, Reisensohn C, Kewer JL, et al. Effects of the short-stitch technique for midline abdominal closure: short-term results from the randomised-controlled ESTOIH trial. Hernia. 2022;26(1):87-95.

3. Millbourn D, Cengiz Y, Israelsson LA. Effect of Stitch Length on Wound Complications After Closure of Midline Incisions: A Randomized Controlled Trial. Archives of Surgery. 2009;144(11):1056-9.
